# Easily Established and Multifunctional Synthetic Nanobody Libraries as Research Tools

**DOI:** 10.3390/ijms23031482

**Published:** 2022-01-27

**Authors:** Bingying Liu, Daiwen Yang

**Affiliations:** Department of Biological Sciences, National University of Singapore, 14 Science Drive 4, Singapore 117543, Singapore; liubingying@u.nus.edu

**Keywords:** nanobody, synthetic nanobody library, nanobody library design and construction, protein structure determination, protein detection, protein purification

## Abstract

Nanobodies, or VHHs, refer to the antigen-binding domain of heavy-chain antibodies (HCAbs) from camelids. They have been widely used as research tools for protein purification and structure determination due to their small size, high specificity, and high stability, overcoming limitations with conventional antibody fragments. However, animal immunization and subsequent retrieval of antigen-specific nanobodies are expensive and complicated. Construction of synthetic nanobody libraries using DNA oligonucleotides is a cost-effective alternative for immunization libraries and shows great potential in identifying antigen-specific or even conformation-specific nanobodies. This review summarizes and analyses synthetic nanobody libraries in the current literature, including library design and biopanning methods, and further discusses applications of antigen-specific nanobodies obtained from synthetic libraries to research.

## 1. Introduction

**Figure 1:**
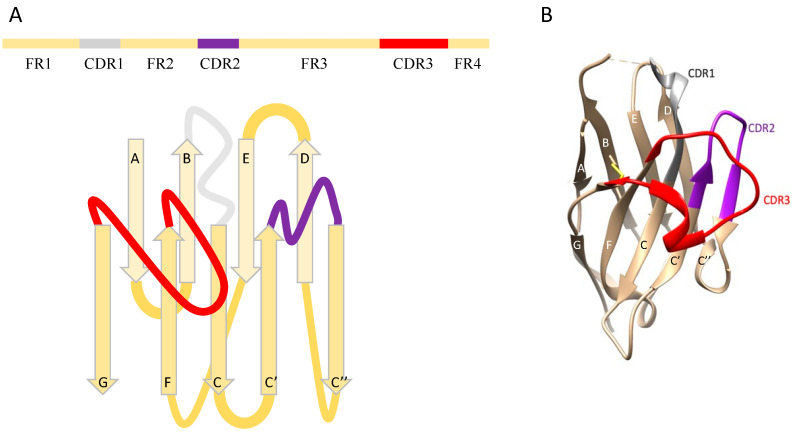
Schematic representations of nanobody architecture and structure. (**A**) Nanobody is composed of four framework regions (FR1-4) and three hypervariable regions (CDR1-3). The structural architecture of nanobodies includes 2 β-sheets, one with 4 β-strands (A, B, D, and E) and one with 5 β-strands (C, C’, C”, F, and G). CDR1, CDR2, and CDR3 are labeled by grey, purple, and red, respectively. (**B**) Structure of CabBCII-10 nanobody (PDB ID: 3DWT). CDR1, CDR2, and CDR3 are labeled by grey, purple, and red, respectively. A conserved disulfide bond between Cys23 and Cys94 is shown in yellow.

Conventional heterotetrameric antibodies are composed of two heavy chains and two light chains. In Camelidae, a type of antibody is composed solely of heavy chains, but lacks light chains and the first constant domain (CH1) of conventional heavy chains [1]. The heavy chains from heavy-chain antibodies (HCAbs) comprise two constant domains (CH2 and CH3) and one antigen-binding domain that is termed nanobody, or VHH, or single-domain antibody. Nanobodies are functionally equivalent to conventional antibodies’ antigen-binding fragments in recognizing target antigens. Their molecular weights are around 12 to 15 kDa, about one-tenth of conventional antibodies’ size. Nanobodies have four framework regions (FR1-4) and three hypervariable regions (CDR1-3). The structural architecture of nanobodies comprises 2 β-sheets, one with 4 β-strands and the other with 5 β-strands, and CDRs form flexible antigen-binding loops between β-strands [2] (Figure 1). CDRs are responsible for recognition and binding. CDR3 is the dominating contributor, while CDR1 and CDR2 assist in the binding. Compared with conventional antibodies, nanobodies have longer CDR3, which provides them with more diverse paratopes [3]. For example, nanobodies can interact with enzymes by entering the clefts of catalytic sites [4]. Moreover, nanobodies exhibited excellent binding affinities with low nanomolar or even picomolar K_D_ (equilibrium dissociation constant) values [5]. The characteristics of small size and high specificity mean that nanobodies are suitable for many more applications than conventional antibodies. The most successful application is Caplacizumab—the first FDA-approved nanobody-based drug [6]. Besides the therapeutic application, which requires complex engineering and optimization steps, nanobodies can be used as research tools. One of the wide usages of antibodies in research is specific protein detection such as Western blot and ELISA. Nanobodies are fully qualified to substitute antibodies in assisting research and have more potential usages as their size is much smaller.

The identification of antigen-specific nanobodies is highly reliant on the construction of nanobody libraries. In the same manner as antibody libraries, there are three types of nanobody libraries—immune, naïve, and synthetic libraries—which can be applied to retrieve antigen-specific nanobodies. The immune library is generated by immunizing animals, such as dromedary, llama, or transgenic mice producing HCAbs [7,8], and subsequently harvesting the host’s blood. After that, mRNAs in lymphocytes are converted into cDNAs and used to amplify the nanobody genes. The main advantage of immune libraries is that high binding-affinity nanobodies can be obtained. In vivo maturation of nanobodies makes immunization libraries the most widely used. At the same time, disadvantages include multiple libraries being needed for different antigens; non-immunogenic antigens, or antigens with an adverse effect on hosts which may fail to induce an immune response; and the high cost of animal immunization. For generating a naïve library, peripheral blood lymphocytes are taken from non-immunized donors. The naïve library avoids the immunization step and takes advantage of the high diversity of the host’s immune system. Because there is no affinity maturation step, such as in vivo immunization, a large pool of blood is required to produce a large library size. The affinity improvement step might be needed to obtain high binding-affinity nanobodies [9]. Moreover, accessibility to Camelidae might be difficult for most biological laboratories.

Nevertheless, the urgent need for antigen-specific binders is a common issue that a majority of laboratories encounter. A synthetic nanobody library is an easily established and multifunctional platform to obtain binders for different antigens once a laboratory can achieve molecular cloning. Published works based on synthetic nanobody library are minimal (Table 1). These works have clearly demonstrated the approaches about the construction and selection of antigen-specific nanobodies but have not unraveled the broad application of synthetic nanobody libraries. This review will summarize the methods for constructing and biopanning synthetic nanobody libraries and further discuss applications of antigen-specific nanobodies selected from such libraries in research.

**Table 1:** Overview of the published synthetic nanobody libraries with their frameworks, CDR randomization designs, selection methods, antigens, and applications [10,11,12,13,14,15,16,17].

Library Name	Framework	Randomized Region	Randomized Region Design	Biopanning Method	Antigen	Application
Ju library [10]	h_NbBcII10_FGLA_	3 CDRs	Analyzed sequences of nanobodies found in nature and chose amino acids to enhance hydrogen bonding and hydrophobic interactions	Phage display	Interleukin-1β (IL-1β), Amyloid-β, Vascular endothelial growth factor (VEGF)	Identified nanobodies recognizing IL-1β, amyloid-β, and VEGF
Yan library [11]	cAbBCII10	CDR3 only	NNK (where N = A/T/C/G, and K = G/T)	Phage display	Human prealbumin (PA), Neutrophil gelatinase-associated lipocalin (NGAL)	Developed a PA detection system
Wang library [12]	cAbBCII10	3 CDRs	NNK	Phage display	Glypican-3 (GPC3)	Identified four anti-GPC3 nanobodies as potential molecules for HCC diagnostic and therapeutic drugs
Wei library [13]	cAbBCII10	3 CDRs, and the length of CDR3 (9–20 amino acids)	CDR1+CDR2: partially randomization; CDR3: NNK	Phage display	M2 ion channel protein of influenza A virus	Showed potent neutralizing activities of nanobodies for influenza A viruses
NaLi-H1 [14]	hs2dAb	3 CDRs, and the length of CDR3 (9, 12, 15 or 18 amino acids)	Analyzed the natural diversity from a llama naïve library. CDR1+CDR2: partially randomization; CDR3: fully randomization except for cysteine	Phage display	βActin, Tubulin, EGFP, mCherry	Selected nanobodies fused to a proteasome-targeting domain showed specific degradation of their targets and can be a potential tool for rapid protein knockdown in both cells and animals.
McMahon library [15]	a consensus framework derived from llama genes IGHV1S1-S5	3 CDRs, and the length of CDR3 (10, 14, or 18 amino acids)	Recapitulated the diversity of nanobodies uploaded in the wwPDB database	Yeast display	Human serum albumin, Metabolic hormone adiponectin, β2 adrenergic receptor, Human A2A adenosine receptor	Established an in vitro platform to choose conformationally selective nanobodies
Sevy library [16]	Alpaca IGHV3S53 and its humanized version	3 CDRs, and the length of CDR3 (6–18 amino acids)	Mimicked the natural occurring VHH repertoire	Yeast display	Soluble mouse Programmed cell death protein 1(PD-1) ectodomain, Amyloid-β peptide, A G-protein coupled receptor (GPCR)—MrgX1	Used anti-mPD-1 nanobodies to block mPD-1 and mPD-L1 interaction
Zimmermann library [17]	3 nanobodies in RCSB PDB database: 3K1K, 3P0G, 1ZVH	3 CDRs, and the length of CDR3 (6, 12, or 16 amino acids)	Obtained a balance between charged, polar, aromatic, and apolar amino acids, and based on the location of different structures such as in loops, in the middle of β-sheets	Ribosome display and subsequent phage display	Maltose-binding protein (MBP), Bacterial ABC transporter IrtAB and TM287/288, Human Solute Carrier (SLC) transporter ENT1 and GlyT1	Recognized nanobodies targeting the transient ATP-bound state of bacterial ABC transporter TM287/288; Generated conformational-selective nanobodies against flexible transporters ENT1 and GlyT1

## 2. Synthetic Nanobody Library Design

**Figure 2:**
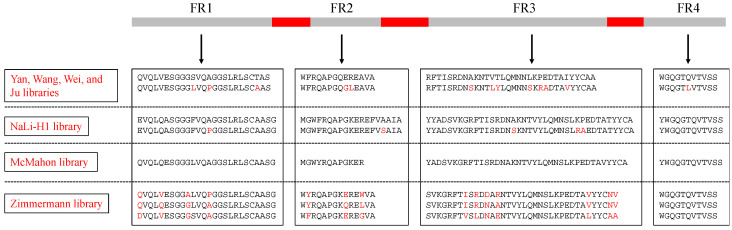
Protein sequences of nanobodies’ framework. The Yan, Wang, Wei, and Ju libraries used cAbBCII10 and its humanized version (h-NbBCII10_FGLA_). Amino acids with red color indicate humanized positions. The NaLi-H1 library used sdAb^D10^ humanized version (hs2dAb). Amino acids with red color indicate humanized positions. The McMahon library used a consensus framework derived from llama genes IGHV1S1-S5. The Zimmermann library used three scaffold sequences corresponding to three different CDR3 lengths. The three sequences from top to bottom represent three groups—concave (six amino acids for CDR3), loop (12 amino acids for CDR3), and convex (16 amino acids for CDR3). Amino acids with red color indicate the non-conserved residues among these three scaffolds.

When a synthetic nanobody library needs to be constructed, a proper framework sequence should be determined. Unlike nanobodies from the animal host, each having its own framework region, all nanobodies from a synthetic nanobody library need to share the same framework region sequence. The shared scaffold needs to be stable, well-expressed, and highly-universal. A natural nanobody has been tested as a good option for synthetic nanobody libraries’ scaffold, named cAbBCII10 (Figure 2). cAbBCII10 has been identified as a plastic framework that allows the successful exchange of antigen specificities from donor nanobodies to its framework. Moreover, unlike other nanobodies, which require a conserved disulfide bond to fold correctly, cAbBCII10 can fold into a functional structure even without the conserved disulfide bond, which is crucial for intrabodies to be applicable in the intracellular reducing environment [18]. Humanization of this framework was also conducted to minimize immunogenicity to humans [19]. Some relatively early published synthetic nanobody libraries such as the Yan, Wang, Wei, and Ju libraries preferred using this framework or the humanized version as research on the universal nanobody scaffold was very limited [10,11,12,13]. To choose the scaffold for the NaLi-H1 library, Moutel et al. screened several hundred clones from immune or naïve llama VHH libraries to find a nanobody with excellent solubility and stability [20,21]. Nanobodies from llamas usually only have one disulfide bond, which are more suitable for identifying universal scaffolds [21]. They applied a chloramphenicol filter assay to exclude those easily aggregated or easily degraded nanobodies. This assay was based on the fusion of HA-tagged chloramphenicol acetyl transferase (CAT) to the C-terminal of nanobodies. Only functional fusions of nanobodies and CAT in the bacterial cytosol could degrade a high concentration of chloramphenicol and survive. They further analyzed those nanobodies’ expression levels and solubility as EGFP fusions in the mammalian cell cytoplasm. The identified nanobody’s framework sequence matched the sequence of the most robust llama’s nanobody framework, represented by the anti-EGFR nanobody D10 [22], which was determined as the scaffold for the NaLi-H1 library [14] (Figure 2). This nanobody was further humanized based on the human VH3 sequence while retaining the VHH-specific amino acid hallmarks essential in increasing solubility. Besides screening for a universal nanobody framework, which requires lots of work, the McMahon library used a consensus framework derived from llama genes IGHV1S1-S5 (Figure 2), a simple and practical method for obtaining a stable scaffold [15]. Instead of using only one universal framework, the Zimmermann library proposed a strategy to determine the framework sequence based on the length of CDR3. It classified nanobodies into three groups based on the CDR3 length—concave, loop, and convex, as they thought the long CDR3 needed to be tethered by an extended hydrophobic core. For each group, they searched for a structure-solved nanobody as the framework template [17] (Figure 2). In this way, it could be better to mimic the surface complementarity repertoire of immune libraries. Still, three different libraries need to be constructed instead of one, and the chosen framework’s universality is unclear.

After choosing the framework sequence, design of CDRs’ randomization is the next step. The diversity of variable regions cannot be infinite, so balance between diversity and effectiveness is the primary concern when creating a synthetic library. The diversity means randomizing the hypervariable regions as much as possible, including the number of CDRs and the length of each CDR. CDR3 is the dominant region that determines binding affinity and specificity so that the library can be designed based on randomization of CDR3 only or three CDRs simultaneously. Besides the number of CDRs, the length of each CDR can be varied. The CDR3 lengths of natural nanobodies vary from three to 28 amino acids [23], and for nanobodies with one and two disulfide bonds, the average lengths are 14 and 17 amino acids, respectively [24]. For example, the NaLi-H1 library used several typical CDR3 lengths such as 9, 12, 15, and 18 [14]. Effectiveness means limiting the randomization of CDRs under reasonable scenarios as much as possible. The simplest way of achieving the randomization is to use degenerate codon NNN or NNK (where N = A/T/C/G, and K = G/T), which does not require codon design for various amino acids as NNN or NNK can cover all the 20 amino acids. However, it is impossible to reach the astronomical sequence diversity, and blindness of the library composition leads to less effectiveness. The Yan library and Wang library used this strategy to select anti-human prealbumin (PA) and anti-glypican-3 nanobodies, respectively. The Yan library used a long CDR3 with 16 amino acids and randomized CDR3 only [11], while the Wang library randomized three CDRs, with 8, 8, and 16 amino acids, respectively [12]. The respective theoretical diversities were over 10^28^ and 10^38^, but the achieved diversity of these libraries was around 10^9^. This simplest randomization method is acceptable to identify nanobodies without any requirement in affinity using standard experiments.

**Figure 3:**
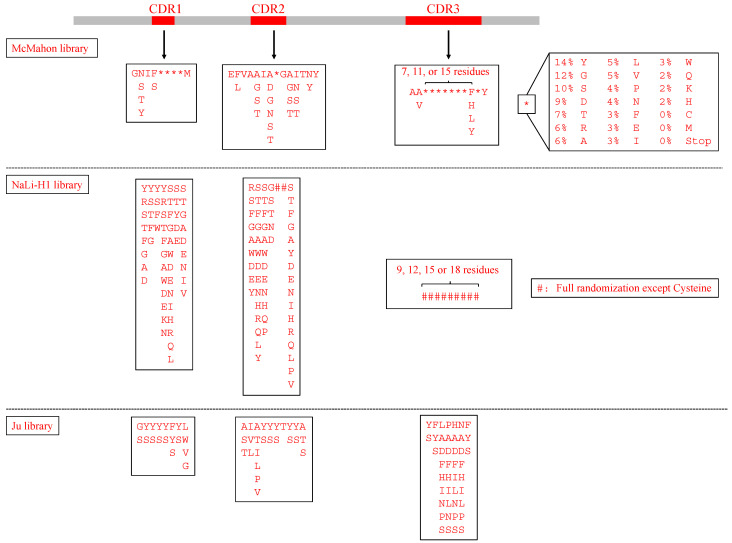
CDR sequence design of three nanobody libraries. The McMahon library tried to recapitulate the position-specific variations in the CDRs from the wwPDB database. The partial randomization positions allowed only a few highly observed amino acids, while the full randomization positions allowed 18 amino acids with different frequencies. Moreover, the length of CDR3 included 10, 14, and 18 residues. The NaLi-H1 library managed to recapitulate the position-specific variations in the CDRs from a naïve llama nanobody library. It introduced four different lengths of CDR3 and full randomization for all the residues in CDR3. The Ju library used short CDR3 and limited randomization to restrict the diversity in a manageable size.

Besides complete randomization for simple scenarios, degenerate codons can be designed based on some criteria. Natural occurrence is the most widely used criterion. Due to lack of a well-established and comprehensive nanobody database, some research groups tried to build nanobody databases. For example, SAbDab-nano is a database that contains all nanobodies available in the wwPDB database with further processing and annotation [25], and iCAN database includes the sequences and structural information about nanobodies from the wwPDB database, EMBL, PubMed, and public patents [26]. By aligning sequences from the databases, the occurrence of each amino acid at the variable regions can be counted. Following the natural occurrence, the variety decreases to a comparatively reasonable size and is constrained to a highly reasonable scenario in which improper folding, aggregation, and low solubility are limited. Nanobodies from the wwPDB database have been used as references for the design of CDRs as these nanobodies with already known structures have the properties of high stability and solubility. The McMahon library analyzed 93 unique sequences from the wwPDB database to obtain position-specific diversity in the CDRs and tried to recapitulate that diversity. For moderately variable positions, they introduced partial randomization, which allows amino acids with high occurrence frequencies at these positions only. More thorough randomization was introduced for highly variable positions, excluding cysteine and methionine to avoid chemical reactivity [15] (Figure 3). The NaLi-H1 applied the same design by analyzing 250 llama VHH sequences isolated from a naïve library and recapitulating the CDRs’ diversity [20] (Figure 3). Although these libraries followed the natural occurrence, they introduced at least 18 amino acids (excluding cysteine and methionine) at one position in the highly variable regions, which leads to a diversity far beyond the achievable variety (around 10^9^). Unlike other libraries with a huge gap between the designed and achieved varieties, the Ju library tried to constrain the designed variety to a manageable size using phage display. The designed variety was around 10^11^ by using short-length CDR3 containing seven amino acids and limiting amino acid diversity within three different amino acids at moderately variable positions and within nine at five highly variable positions [10] (Figure 3). Libraries with a manageable diversity are designed to allow complete control of the library contents, while libraries with a higher designed diversity can introduce more opportunities to find high-binding affinity nanobodies.

Besides following natural occurrences, a synthetic library can be designed to recognize specific antigens based on the already known epitopes [27,28]. Still, there is no synthetic library specifically designed for one antigen or one type of epitopes, as a significant advantage of synthetic libraries is its broad applicability to various antigens.

## 3. Construction of Synthetic Nanobody Library

**Figure 4:**
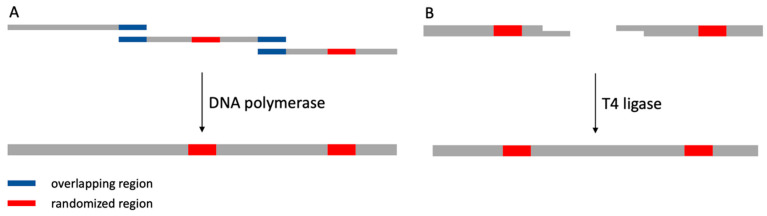
Illustration of overlapping PCR and ligation method to assemble different fragments to full-length nanobody DNA. (**A**) Overlapping PCR uses overlapping regions among different primers to assemble short fragments into long, full-length nanobody DNA. (**B**) Short fragments with compatible ends can be ligated by T4 ligase to generate long, full-length nanobody DNA.

Based on the design, synthesis of enough DNAs with different sequences is essential for constructing the library. Although companies offer high-quality DNA libraries’ construction services, the more economical way is by overlapping PCR (Figure 4A). The overlapping PCR uses several overlapping regions in primers to assemble them as a longer DNA [29]. The only difference between overlapping PCR and regular PCR is the lack of a template. The full-length nanobody genes can be divided into several fragments, and each fragment can be purchased as a primer. Each primer needs to contain an overlapping region for assembly. When the primer is ordered as a randomized version, the primer-synthesis company will add mixed bases at each specific site. Moreover, the percentage of each nucleotide at a position can be customized, which is quite helpful in building a library with specific occurrences for different amino acids. Then these primers can be assembled by overlapping PCR through the overlapping regions at the primer’s 5′ or 3′, and the final product is a mixture of DNAs with different nucleotide sequences at randomized positions. Besides the use of overlapping PCR only, the Zimmermann library used T4 ligase to ligate two DNA fragments with compatible ends (Figure 4B). For a library with different lengths of CDR3, each length can be prepared as a mixed pool, and these pools can be mixed further with a specific ratio later. The final products can be ligated into different plasmids depending on the biopanning methods.

Besides the sufficient quantity required to build a library, quality is another critical issue. An undesirable frameshift mutation is common in synthetic nanobody libraries, which leads to poor library quality. The possible reasons include: (1) the quality of primers is poor as the coupling efficiency during DNA synthesis cannot reach 100%, so that the full-length product decreases as the sequence length increases; (2) the fidelity of DNA polymerases cannot reach 100%; (3) DNA might be damaged during the process of gel extraction and purification. Once primer quality, DNA polymerase fidelity, and experimental techniques are optimized, the quality of the library is not a big problem, even though it cannot reach 100% accuracy. To further minimize the error rate, some libraries applied an extra in-frame selection step. The Ju library developed a growth-based selection of in-frame nanobody sequences, which fused a β-lactamase gene to the C-terminus of the nanobodies’ genes [10]. In this way, only clones expressing functional β-lactamase could survive in a high ampicillin-concentration environment. Nonetheless, the in-frame selection plasmid is different from phagemid for the subsequent phage display, which introduced an additional step of double digestion and re-ligation. Because of the extra workload, most of the synthetic libraries did not perform the in-frame selection step.

## 4. Biopanning Method

Several biopanning methods have been developed to retrieve antigen-specific nanobodies from libraries. “Nanobody” will be used in this review to reduce redundancy, but these biopanning methods can also be applied to antibodies, and antibody fragments. Biopanning methods aim to link a genotype to a phenotype to allow for screening of nanobody libraries. The most widely used one is the phage display. The filamentous bacteriophage is the mainly used phage system. At the early development stage of phage display, nanobody genes were fused to the N-terminus of g3p or g8p in the phage genome. Such fusion leads to a decrease in infectivity and limits the displaying library size [30]. Phagemids were introduced to overcome these limitations. Phagemids encode an antibiotic resistance marker, a signal peptide, and the phage coat protein g3p or g8p [31]. G8p is the major coat protein which is present in around 2700 copies while g3p is a minor coat protein which is present in 3 to 5 copies. Proteins or peptides fused with g8p are presented at high valency, which permits selection of very low affinity ligands due to the increased avidity. Moreover, to preserve g8p’s functionality, g8p is preferred to fuse with short peptides. Both peptides and folded proteins can be fused with g3p and low valency of g3p fusions limits the selection to high affinity, which is essential in biopanning a nanobody library to retrieve high affinity binders [32]. The nanobody sequence is cloned at the 5′ of the g3p or g8p coat protein sequence, and its expression is controlled by a promoter such as lacZ. Phagemids are around 4600 bp in size, relatively smaller than the phage genome, leading to higher transformation efficiency [30]. Despite the higher transformation efficiency, the variety of a library using phage display needs to be constrained within 10^10^ because of the limitation of transformation efficiency. As phagemids alone cannot produce infective phages, a helper phage containing the genes for phage replication and assembly is required to infect the cells with phagemids. After the helper phage genome is incorporated into the cell, phage production that displays g3p- or g8p-nanobody commences. To increase the percentage of phages packaged with phagemid, the genome of helper phages possesses a modified IG (intergenic region) that is replicated and packaged less efficiently than wild-type IG in phagemid [33]. Once the phages displaying g3p- or g8p-nanobodies are assembled, phages displaying anti-antigen nanobodies can be selected by several biopanning rounds using immobilized antigens. Biopanning can be performed on solid surfaces, in solution, or even on cell surfaces [34]. Immobilization antigens on solid surfaces such as polystyrene plates or tubes is robust. The most widely used coating method is through passive adsorption, which is quite direct, but might alter antigens’ conformation [33]. In-solution biopanning can overcome this obstacle through affinity capture of antigens with specific modifications or tags in solution [35]. For some membrane proteins which are quite large and complicated, it is difficult to purify and coat them on solid surfaces. Thus, cell-based biopanning can be utilized to select antigens targeting membrane proteins at their native conformations [36,37]. Phages displaying nanobodies which bind to antigens can be enriched through several rounds of biopanning. These phages will be subsequently picked and screened using ELISA assay to confirm the binding and then sequenced before protein expression and purification [38].

Besides phage display, yeast display and bacterial display are also applicable for screening synthetic nanobody libraries [39,40]. The principle is the fusion of nanobodies with cell surface proteins. The less common usage of these two display methods might be the relatively low transformation efficiency (~10^8^), as synthetic library diversity needs to be at least 10^9^. For yeast display, the typical design is to fuse nanobodies to the C-terminus of yeast cell surface protein AgaII-AgaI so that nanobodies can be displayed on the surface of yeast [41]. Instead of using an engineered yeast strain that expresses galactose inducible AgaI-AgaII, the McMahon library established a simplified system that replaced linker protein AgaII-AgaI with a single tether and reached a diversity of 5 × 10^8^ [15]. Nanobodies can be fused to different proteins for bacterial display based on the type of bacterial hosts, such as Gram-positive or Gram-negative. *E.coli* is suitable for bacterial display because of its high transformation efficiency, fast growth, and well-established expression and secretion system. An intimin fusion system was optimized for *E.coli* display of nanobodies. Nanobodies were in frame with N-terminal fragment of intimin, and one cell could display ~8000 nanobody molecules [42]. The bacterial display has been used to select antigen-specific nanobodies from the immune library but not from the synthetic library so far [43]. Nonetheless, one of the most significant advantages of yeast or bacterial display is the combination with flow cytometry, which monitors the selection process. In the meantime, yeast and bacterial displays are multivalent and less sticky than the phage display, which is more suitable for selecting antigens on complex structures such as cell surfaces [39,41].

All the biopanning methods mentioned above include a transformation step, which restricts the diversity of the synthetic nanobody library within 10^10^. The main restrictions of transformation efficiency include the quality and quantity of competent cells, the amount and purity of DNA used, the electrical parameters for electroporation, and so on [44]. The transformation step of the NaLi-H1 library was described in a detailed way, which was quite representative. It followed the most optimized protocol and used commercial TG1 cells from the Lucigen. After performing 20 electroporations with around 400 ng ligation products each time, 3 × 10^9^ individual clones were obtained [14]. This example shows the restriction of the transformation step in limiting the achievable variety of synthetic nanobody libraries. Ribosome display omits this step and leads to higher diversity [45]. The ribosome display of nanobodies is through fusion library genes to a spacer sequence lacking a stop codon. When translated, the spacer sequence attaches to the ribosome while the translated part—the nanobodies—can be used for the biopanning step [46]. The Zimmermann library used ribosome display to perform the rough screening with a library diversity around 10^12^ and phage display subsequently to enrich the pool of positive binders further. Although the transformation is no longer the restriction step, the diversity cannot increase too much as the ligation step used to attach spacer sequences to nanobody genes is another bottleneck [17]. The limited usage of ribosome display is due to the requirement of RNA-related experiments, which is a hurdle for non-expert laboratories. Fortunately, commercial in vitro translation kits such as PUREfrexSS (GeneFrontier, Kashiwa, Japan) are available to allow for more laboratories to use ribosome display.

## 5. Nanobody Application in Research

The identified antigen-specific nanobodies can be widely used in research. To date, most of the nanobodies used to assist research are from immunization libraries. The most straightforward application is protein purification and recognition. Common protein tags such as His-tag can be fused with nanobodies to immobilize the nanobodies, and tags are preferably linked at the C-terminal of the nanobodies, which allows for the paratope to orient toward the solvent [47]. Anti-GFP and anti-YFP nanobodies immobilized to Sepharose resin have been used to facilitate the purification of lowly expressed GFP and YFP fusions from mammalian cell lysate [48]. Moreover, antigen-specific nanobodies can be fused to alkaline phosphatase or horseradish peroxidase used in ELISAs, and they have been successfully used in virus detection and toxic material detection [49,50,51]. The small size and high specificity of nanobodies allow for the very limited adherence to non-specific proteins. A nanobody against PA was identified from the Yan library and subsequently a PA-ELISA detection system was built by immobilizing the nanobody on a 96-well plate to capture PA. This system could detect PA in a wide range of concentrations from 50 to 1000 ng/mL [11].

In addition to protein detection and purification, another significant application of nanobodies is the structural characterization of their antigens. For highly dynamic proteins such as amyloidogenic proteins and some membrane proteins, their structures are difficult to be determined as they are in equilibrium among different conformations. One of the advantages of synthetic nanobody libraries is the ability to control antigen concentration and conformation as the antigens are displayed in vitro. The interaction of a nanobody and its antigen can reduce antigen’s motion and fix it in a preferred conformation. Structure determination of G-protein-coupled receptors (GPCRs) at active state created a big challenge as GPCRs need specific signaling partners to be stable at a fully active state. Some conformation-specific nanobodies serving as G protein mimetics have been used to obtain active-state structures of β1 and β2 adrenergic receptors [52,53]. Before 2018, all nanobodies targeting GPCR were from immunization libraries. With the increasing awareness of the broad applicability of synthetic nanobody libraries, several libraries have been constructed to establish the process of screening for conformation-specific nanobodies [15,17]. The McMahon library’s authors used yeast surface display and identified nanobodies targeting agonist occupied human GPCR β2AR and A_2A_R. The Zimmermann library’s authors used a combination of ribosome display and phage display to find nanobodies targeting conformation-specific membrane proteins. So far, over 150 nanobody–antigen co-crystallization structures have been uploaded to the wwPDB database [3]. As more and more studies focus on synthetic nanobody libraries, the number of nanobody–antigen co-crystallization structures will increase dramatically in the near future.

In addition to crystallization, the small size of nanobody makes it more versatile for nuclear magnetic resonance (NMR) spectroscopy than conventional antibodies, as antibodies or even antibody fragments are too large, which hinder the analysis of antibody–antigen complexes by solution state NMR. Nanobody binding can cause a detectable change in the tumbling rate of their antigen while retaining the possibility to obtain high-resolution NMR spectra [54]. NMR has been successfully used to illustrate the dynamics of ATP7B, a transmembrane copper transporter powered by ATP hydrolysis. The N-terminal domain of ATP7B consists of six metal-binding domains (MBD1-6), and Huang et al. clarified their role in ATP7B trafficking by using nanobodies to detect the tumbling rate change of different domains. The nanobody bound to MBD3 slowed its target domain’s tumbling rate while leading to faster tumbling rates of MDB1 and MDB2, which indicates that the interactions among these three domains are broken by this nanobody. In contrast, the nanobody bound to MBD4 only decelerated its target domain. Combining with studies in cells, it was found that disruption of the domain–domain interactions in MBD1-3 produced an open conformation that triggers ATP7B trafficking [55].

Recently, nanobodies have been used in the field of Cryo-EM for protein structure determination. In the same manner as the application as a conformational fixing agent in crystallography, nanobodies have also been used to solve the cryo-EM structure of adenosine A_2A_R coupled to an engineering heterotrimeric G protein composed of mini-GS-β1γ2 and a conformationally selective nanobody that binds to G proteins to stabilize its complex with activated GPCRs [56]. More recently, Uchański et al. grafted nanobodies onto larger protein scaffolds termed megabodies to increase their molecular weight while retaining the binding affinity and specificity [57]. Megabodies help overcome major obstacles that limit cryo-EM reconstructions’ high resolution, such as small particle size and nonrandom orientation at the water–air interface [58]. The structure determinations of membrane proteins have suffered from severe preferential orientation in amphipols or nanodiscs. Uchański et al. used GABA_A_R-specific megabodies to interact with the GABA_A_R β3 subunit. More than 77% of the classified particles showed different orientations, resulting in a high-quality 2.49 Å resolution 3D reconstruction. They further developed megabodies that bind to nanodiscs prepared by using membrane scaffold proteins (MSPs) such as MSP1D1, MSP1E3D1, and MSP2N2. Such megabodies help to randomize the orientation of nanodiscs-embedded membrane proteins, and successfully reconstructed a serotonin 5-HT3A receptor in a lipid bilayer [57].

Apart from in vitro usage of nanobodies, intracellular expression of nanobodies also has many applications. The first innovative tool is chromobodies constructed by fusing nanobodies with fluorescent proteins to generate intracellular fluorescent, antigen-recognition nanobodies. Chromobodies can be expressed in living cells and used to recognize and trace antigens. They have been successfully used to localize cellular compartments such as cytoskeletal actin filaments and histone proteins for chromatin labeling [59]. Furthermore, nanobodies have been used for super-resolution imaging. Super-resolution microscopy always requires photostable labeling, while chromobodies suffer from photobleaching during extensive image acquisition. To overcome this limitation and better cooperate nanobodies with super-resolution microscopy, nanobodies conjugated with organic dyes were developed and became attractive probes for super-resolution microscopy [60]. Ries et al. applied nanobodies conjugated with organic dyes to achieve nanometer spatial resolution of microtubules. The full-width half maximum of individual microtubules was 26.9 ± 3.7 nm (s.d.) using AF647-anti-GFP nanobodies to stain tubulin-YFP. This result was compatible with the reported microtubule diameter (~25 nm), which was much closer than the results from anti-GFP or anti-tubulin antibodies. This dominance is due to the small size of nanobodies, which leads to minimal linkage error when conjugating with other molecules [61].

Besides determination of biomolecule localization, nanobodies inside cells can also be used as a protein knockout tool. A method called deGradFP can be used to quickly deplete GFP fusions in the eukaryotic system by fusing an anti-GFP nanobody gene to an F-box domain in *Drosophila*. Once the nanobody binds to its antigen, the antigen will be polyubiquitinated and lead to degradation [62]. The NaLi-H1 library’s authors imitated this application by using several anti-EGFP nanobodies and fusing them to the F-box domain. Although not all nanobodies fused with F-box domain could be functional inside cells, several nanobody and F-box domain fusions showed complete deletion of EGFP fusion proteins [14].

## 6. Conclusions

Synthetic nanobody libraries use cheap DNA oligonucleotides and avoid expensive and complicated animal experiments. This easily established platform provides every laboratory an opportunity to build its own libraries and obtain antigen-specific nanobodies for various applications. Although the number of published synthetic nanobody libraries is limited, they have shown how to create synthetic nanobody libraries and their vast potential as research tools. Several biopanning methods have been used individually or in combination to select antigen-specific nanobodies. These libraries have been used to obtain antigen-specific nanobodies to build antigen-detection systems, identify conformation-specific nanobodies, determine structures of proteins with high mobility, localize antigens, and engineer antigen’s expression level in cells. With the already developed methods, more laboratories can use synthetic libraries as a powerful research tool for novel applications.

## Data Availability

Not applicable.
